# Evaluation of Microleakage in Class II Cavities using Packable Composite Restorations with and without use of Liners

**DOI:** 10.5005/jp-journals-10005-1162

**Published:** 2012-12-05

**Authors:** Rajesh Arora, Ravi Kapur, Nikhil Sibal, Sumit Juneja

**Affiliations:** Assistant Professor, Department of Conservative Dentistry, Government Dental College, Postgraduate Institute of Medical Sciences, Rohtak, Haryana, India; Principal, Department of Conservative Dentistry and Endodontics Maharishi Markandeshwar College of Dental Sciences and Research Mullana, Haryana, India, e-mail: endoauthor@gmail.com; Senior Resident, Department of Conservative Dentistry and Endodontics, Safdarjung Hospital and VMMC, New Delhi, India; Dental Surgeon, HCDS, Government of Haryana, Postgraduate Institute of Medical Sciences, Rohtak, Haryana, India

**Keywords:** Microleakage, Packable composites

## Abstract

The advent of the esthetic era and advances in adhesive technology saw the emergence of resin composite materials. But the problem of polymerization shrinkage remained. This was due to the contraction of the resin during curing inducing internal and interfacial stresses at the tooth restoration interface, leading to gap formation and subsequent micro-leakage. A number of techniques and modifications in the material have been proposed to minimize polymerization shrinkage and microleakage. In this study, the hypothesis that the placement of resin-modified glass ionomer cement (RMGIC) or flowable composite, as liner, beneath the packable composite, on the gingival surface of the tooth [coronal or apical to cementoenamel junction (CEJ)], could reduce the microleakage in class II composite restorations, was tested. Sixty recently extracted noncarious human mandibular molars were used. The teeth were randomly divided into three groups (20 specimens each): Group I (Filtek P60 with RMGIC liner), group II (Filtek P60 with Filtek Z350 liner) and Group III (Filtek P60 without liner). The teeth of each group were further subdivided into two subgroups (equal number of cavities). Subgroup A gingival seat 1 mm occlusal to CEJ on mesial side. Subgroup B gingival seat 1 mm apical to CEJ on distal side. It was concluded that in class II composite restorations gingival microleakage is more at the dentinal surface than on enamel. The use of a flowable composite and RMGIC, as liners, beneath the packable composite, in class II composite restorations, significantly reduces the microleakage when margins are in dentin, but the reverse is true, when the margins are in enamel.

**How to cite this article:** Arora R, Kapur R, Sibal N, Juneja S. Evaluation of Microleakage in Class II Cavities using Packable Composite Restorations with and without use of Liners. Int J Clin Pediatr Dent 2012;5(3):178-184.

## INTRODUCTION

A beautiful smile is the demand of the present hour. The search for an ideal esthetic material for restoring teeth has resulted in significant improvements in their properties and technique of application. Composites and acid-etch technique represent two major advances in esthetic restorative dentistry. Initially, their use, as a posterior restorative material had several limitations in terms of strength, dimensional stability and wear resistance that led to the failure of restorations, such as loss of anatomic form and occurrence of secondary caries.^1^ Further research led to improvements in wear resistance and strength of composites, but the problem of polymerization shrinkage remained. Polymerization shrinkage results due to the contraction of the resin during curing inducing internal and interfacial stresses at the tooth restoration interface, leading to gap formation and subsequent marginal leakage.^1^ Polymerization shrinkage results due to the contraction of the resin during curing and has been reported to be in the range of 3%.^2^ Microleakage is the movement of ions, micro- organisms, fluid and substrates across the tooth restoration interface (Kidd, 1976)^3^ and can cause numerous deleterious effects, such as secondary caries, hypersensitivity of the restored tooth and interfacial staining; eventually leading to pulpal pathology. The problem of microleakage has been largely demonstrated mainly below the cementoenamel junction (CEJ) in several studies^4^ because the bonding to dentin is far more difficult and less predictable than bonding to enamel because dentin is less mineralized, about 75% as opposed to enamel which is 98%. Moreover, dentin has a more complex histologic pattern, such as tubular structure and intrinsic wetness.^5^ A number of techniques and modifications in the material have been proposed to minimize polymerization shrinkage and microleakage. These include changes in filler content, use of expanding resin matrices, use of glass and fiber inserts and modifications in curing techniques like soft curing, dual curing, ramp and delayed curing.^6^ Numerous materials like glass ionomer, self-curing composites and more recently the flowable composites were experimented with as stress absorbing liners.^7^ This hypothesis was based on the principles of incremental build- up and utilizing certain unique properties of the proposed materials.^8^

The aforesaid hypothesis was put to test in this study by comparing the marginal leakage of packable composite resin restorations with margins located 1 mm coronal and 1 mm apical to CEJ, using flowable composite and resin- modified glass ionomer cement (RMGIC) as liners.

## MATERIALS AND METHODS

Materials used in the study (Table 1). Sixty recently extracted noncarious human mandibular molars were used. The teeth were stored in distilled water at room temperature. The teeth were cleaned to remove surface debris and randomly divided into three groups, *viz* groups I, II and III of 20 specimens each (Table 2).

Each specimen was mounted in a ring of polyvinyl chloride (PVC) of 1 inch diameter and filled with plaster of Paris. One hundred and twenty standardized class II box shaped uniform cavities were prepared on the mesial and distal surfaces of each tooth. Each cavity was prepared with a carbide bur (# 245, SS White). After every five preparations, a new bur was used. The occlusal and proximal buccolingual width of the cavity was kept at 3 mm, with an axial depth of 1.5 mm. Gingival seat was placed 1 mm occlusal to CEJ on the mesial surface and 1 mm apical to CEJ on the distal surface, of each tooth. The teeth of each group were further subdivided into two subgroups each having equal number of cavities (Table 3).

All the prepared cavities were rinsed with water from a syringe, for 30 seconds and dried with absorbent paper for 15 seconds, before the restoration of the cavities.

In group I, automatrix was applied that allowed building up of the proximal wall. RMGIC was applied to 1 mm thickness as liner on the gingival seat of the mesial and distal preparations. It was cured for 20 seconds using light cure unit. Etchant gel was applied to the enamel and dentin according to the manufacturer’s instructions for 30 seconds. The specimens were then rinsed with water for 15 seconds and dried for 15 seconds with absorbent paper. Then the first coat of bonding agent was applied to the cavity walls followed by another application after 15 seconds, it was air dried and cured for 20 seconds as per manufacturer’s instructions. The specimens were filled with packable composite. The first increment of 1 mm was placed horizontally and light cured for 40 seconds with the light intensity of 600 to 700 mW/cm^2^ (confirmed with radiometer). The thickness of the increment placed was confirmed with the help of a calibrated probe, by measuring the depth of the walls prior to and after the placement of the composite. The rest of the cavity was filled with the help of oblique layering technique, i.e. using triangular or wedge- shaped increments of 1.5 mm thickness, that contacted only one opposing wall at a time. The second and third increments were cured for an additional 10 seconds. The curing was done from the occlusal aspect with the tip of the curing unit placed as close to the occlusal surface as possible. The restorations were then finished and polished on the occlusal surface.

**Fig. 1:**
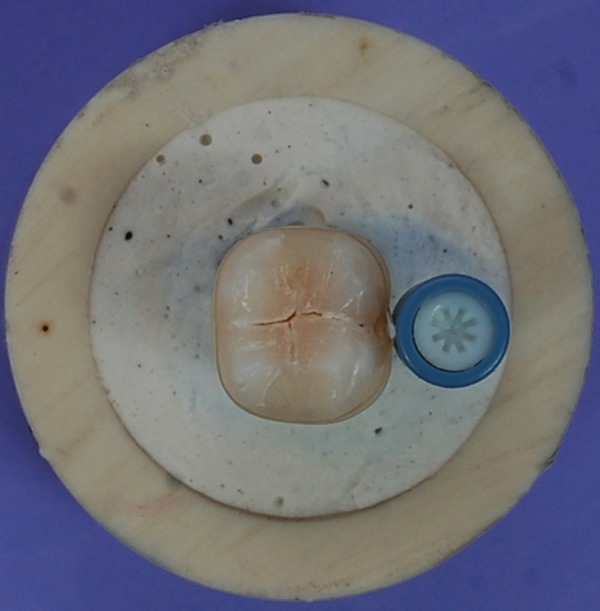
Occlusal view of restored tooth with auto matrix

In group II, the specimens were cleaned with water for 30 seconds and dried with absorbent paper for 15 seconds. Acid etching was done by applying the etchant gel on enamel and dentin surfaces for 30 seconds. It was washed with water for 15 seconds and dried with absorbent paper for 15 seconds. Then the first coat of bonding agent was applied to the cavity walls followed by another application after 15 seconds. It was then air dried and cured for 20 seconds as per manufacturer’s instructions. A cellophane automatrix band was adapted to the tooth and a layer of 1 mm thickness of flowable composite was applied on the gingival seat and light cured for 20 seconds. The cavities were filled with packable composite and finished and polished (Fig. 1). 

**Table 1:** **Table 1:** Materials used

*Materials*		*Company*		*Batch no.*	
Etchant gel (Scotchbond multipurpose etchant)		3M ESPE dental products St Paul, MN		4HY 2008-10	
Bonding agent (Adper single bond 2 adhesive)		3M ESPE dental products St Paul, MN		N130154	
Flowable composite (Filtek Z350)		3M ESPE dental products St Paul, MN		N116073	
RMGIC [GC (Gold Label)]		GC Corporation Tokyo, Japan		0802041	
Glass ionomer light-cured universal restorative material					
Packable composite [posterior restorative material (Filtek P60)]		3M ESPE dental products St Paul, MN		N 145537	
Distilled water		Nice Chemicals Pvt Ltd, Cochin		005119	
Nail varnish					
Basic fuchsin dye 0.5% (Rankam)		Ranbaxy Laboratory			
Normal saline		Baxter, Aurangabad		9104057	
Impression compound		Rolex, Ashoo Sons, Delhi			
Sticky wax		Samit, Delhi			

**Table 2:** **Table 2:** Groups

Group I (Filtek P60 with RMGIC liner)		RMGIC was used as liner with light cure composite restorative material	
Group II (Filtek P60 with filtek Z350 liner)		RMGIC was used as liner with light cure composite restorative material Light cure flowable composite resin was used as liner with light cure composite restorative material	
Group III (Filtek P60 without liner)		Light cure composite was used as a restorative material without the use of any liner	

**Table 3:** **Table 3:** Subgroups

Subgroup A (1 mm occlusal to CEJ)		The gingival seat was placed 1 mm occlusal to CEJ on mesial surface	
Subgroup B (1 mm apical to CEJ)		The gingival seat was placed 1 mm apical to CEJ on distal surface	

In group III, the entire procedure was repeated as explained for group I and II, and the specimens were filled with packable composite and finished and polished.

## EVALUATION FOR MICROLEAKAGE

**Figs 2A to E:**
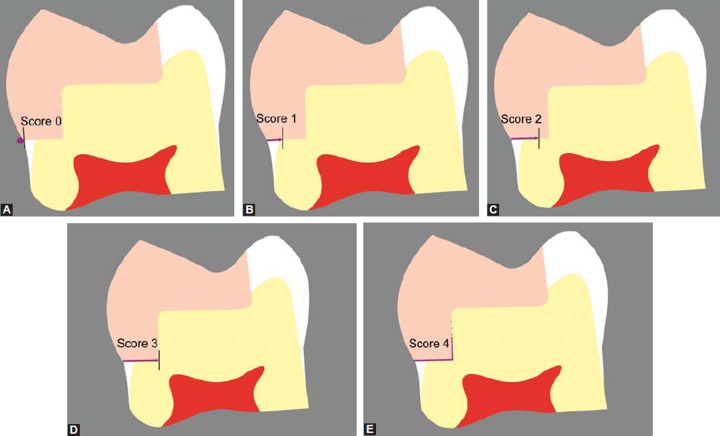
Schematic representation of scoring scale

**Fig. 3:**
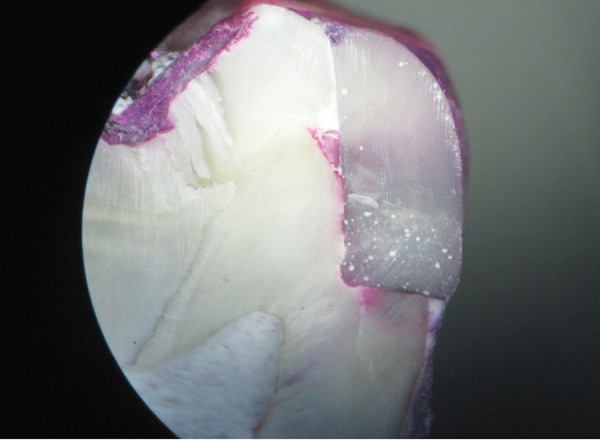
Specimen of group I (RMGIC liner) showing score of 4 on mesial side (dentin margin)

**Fig. 4:**
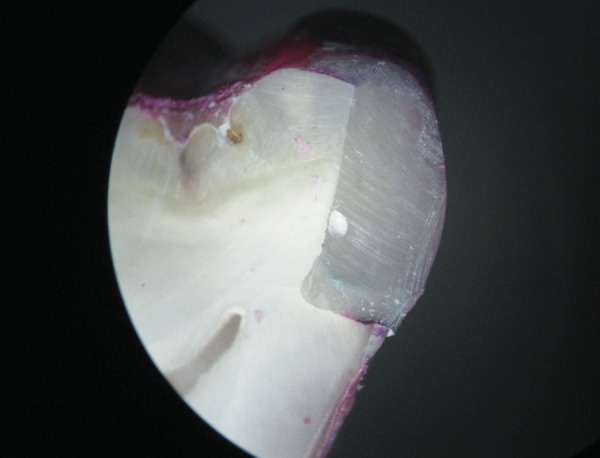
Another specimen of group I (RMGIC liner) showing score of 2 on distal side (dentin margin)

The specimens were thermocycled in water bath for 1,000 cycles between 5°C ± 2°C and 55°C ± 2°C, for dwell time of 30 seconds and transfer time of 10 seconds. Apices of all the teeth were sealed with sticky wax. The surfaces of the teeth, except for 1 mm surrounding the restorations, were coated with two layers of nail varnish. The coated teeth were immersed in 0.5% basic fuchsin dye for 24 hours. The teeth were then sectioned mesiodistally in a vertical plane using a diamond disk revolving at a speed of 20,000 revolutions/sec. The sections were mounted on slides and the degree of dye penetration was recorded under a stereomicroscope (WILD Photomakroskop M400 1,25x, Switzerland) at 40× magnification (Figs 3 and 4). Leakage was evaluated according to scores mentioned for the degree of dye penetration (Table 4 and Figs 2A to E). The readings obtained were recorded for all the three groups and the results were tabulated and submitted to statistical analysis.

## RESULTS AND OBSERVATIONS

**Graph 1:**
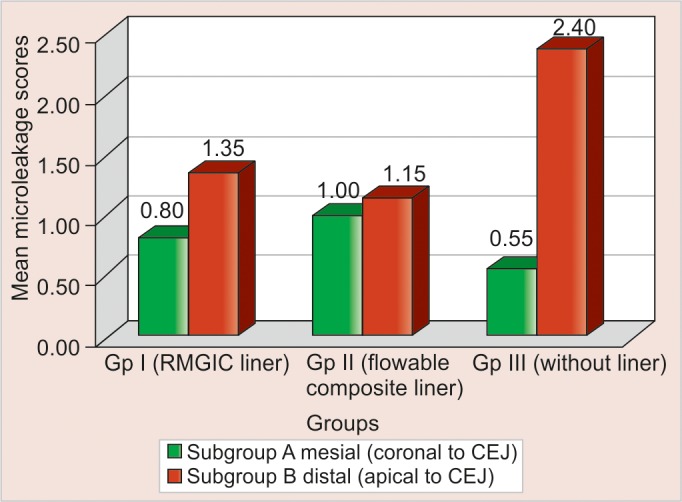
Mean microleakage scores of groups I, II and III with their subgroups A and B

The results and statistical analysis of results are depicted in the form of Graph 1. The Table 5 depict the scores of microleakage, of all the three groups along with their subgroups and their mean values. Since, the microleakage is expressed in terms of scores, nonparametric methods were used for analysis. Kruskal-Wallis test was used for overall comparisons of groups, the Mann-Whitney U-test for individual group-wise comparisons and Wilcoxon Signed rank test for comparing two subgroups of a group.

In this study according to scoring criteria, the more the score, more is the microleakage. Hence, the material showing less microleakage score is better from the clinical point of view.

## DISCUSSION

Polymerization shrinkage occurring during composite curing induces stresses at the tooth restoration interface resulting in gap formation leading to marginal leakage.^9^ In addition to this, the inherent differences in coefficient of thermal expansion between composite resin and the tooth structure also contribute to marginal leakage.^9^ Microleakage is most significant disadvantage associated with the use of composite restorative materials. It is dependent upon several factors including adaptation of resin material to tooth surface, the bonding material used, the technique of bonding, polymerization shrinkage and thermal stability of material. Microleakage may provoke sensitivity due to interfacial hydrodynamic phenomenon and can lead to colonization of microorganisms and high incidence of secondary caries and may clinically cause restoration failure.^7,10^ Restoration of class II cavities with packable composites still remain a controversy with respect to its marginal adaptation in proximal box region and literature has shown that when packable composites are placed apical to CEJ, there is higher marginal leakage from dentin gingival margins, when compared to the cavities placed coronal to CEJ. To overcome this predicament, the use of flowable composite or RMGIC liners in proximal box of class II preparation has been advocated. The flowable composites are less viscous materials due to which it flows easily and adapts well to the tooth surface resulting in less leakage and postoperative sensitivity. They also serve as flexible inter- mediate layer which absorb stress during polymerization shrinkage of composite resin. Light-cured resin-modified glass ionomer are found to be a better alternative to conventional glass ionomer cement liners in reducing marginal leakage at the dentinal margin in class II composite resin restorations.^11^ They reach chemical maturation far more rapidly than conventional glass ionomer cements and resist the occlusal stresses and polymerization contraction of composite.^4^

**Table 4:** **Table 4:** Scoring criteria for microleakage

*S. no.*		*Scoring criteria*		*Score*	
a		No dye penetration		0	
b		Dye penetration up to one-third of the gingival wall		1	
c		Dye penetration up to two-third of the gingival wall		2	
d		Dye penetration up to full length of the gingival wall		3	
e		Dye penetration up to the whole length of the gingival wall and along the axial wall		4	

**Table 5:** **Table 5:** Summary of microleakage score of groups I, II and III along with its subgroups A and B

*Groups*													
		*I*		*II*		*III*		*I*		*II*		*III*	
*Subgroups*													
		*A*		*A*		*A*		*B*		*B*		*B*	
*Score*	*Sample numbers*												
0		11		9		11		5		7		1	
1		5		7		8		6		7		1	
2		2		1		0		6		3		9	
3		1		1		1		3		2		7	
4		1		2		0		0		1		2	
Count		20		20		20		20		20		20	

In order to verify the better technique for minimizing shrinkage, the present study was conducted. According to microleakage score and its mean [group I A (0.80 ± 1.152), group II A (1.00 ± 1.298) and group III A (0.55 ± 0.759)] least microleakage was shown by group IIIA (without liner) followed by group IA (RMGIC liner) and then group II A (flowable composite liner). However, statistical comparative analysis of the mean values showed no significant difference between all these subgroups (p-value = 0.621, NS). Group IIIA showed least marginal leakage values when margins of restoration were placed on enamel. These results are in agreement with the study of Beznos C^4^ who concluded that with enamel margins all the materials and techniques demonstrated a very good seal and very less microleakage, in contrast to the cementum/dentin margins. The probable reasons for the packable composite showing least amount of leakage coronal to CEJ, when margins were placed in enamel may be because packable composite has high depth of cure, low polymerization shrinkage and a bulk fill technique. The bond strength of composite to enamel is usually higher than bond strength of dentin, because dentin is a less favorable bonding substrate and enamel margins of composite restorations are reported as having less leakage than the cementum/dentin margins.^12^ The present results for enamel margins confirm the effectiveness of the enamel etching technique in controlling the microleakage in the gingival wall of a class II composite restoration.^13^

However, the results of Optadam and Roeters^14^ do not concur with those of our study. They concluded from their study that packable composite resin did not show good result even with margins located in enamel, while this has concluded that some of primers could affect the adhesion between bonding agent and the enamel.^14^ Group IA (RMGIC liner) shows more microleakage than group IIIA (without liner). It indicates that placing the RMGIC as a liner does not affect the microleakage, when margins were placed in enamel. However, statistical analysis showed that the difference between two were insignificant (p-value = 0.694, NS). These results were in agreement with study of Dietschi D et al^15^ who concluded that when the glass ionomer cement or resin-modified glass ionomer material were applied as a lining, increased leakage and downgraded marginal adaptation had occurred.

Group IIA (flowable liner) showed highest micro- leakage. These results were in agreement with study of Chuang SF et al who concluded from their study that the use of flowable composite resin liner revealed no significant difference in microleakage between pairs with and without the flowable lining when margins of the restorations were placed in enamel. A flowable composite lining in class II resin filling could effectively reduce voids in the interface and total number of voids in restoration. However, there was no significant correlation between number of restoration voids and associated microleakage. Manufacturer’s data available on P60 reported volumetric change during polymerization shrinkage of approximately 1%, while flowable composite (Filtek Flow) shrinks by about 4%. The increased polymerization shrinkage of the flowable liner may have caused the greater leakage seen in association with these materials.

The second part of study, comprised of restorations of the cavities on distal side (subgroup B-margins placed apical to CEJ). Comparison among various groups showed highly significant difference (p-value = 0.001, HS).

**Flow Chart 1:**
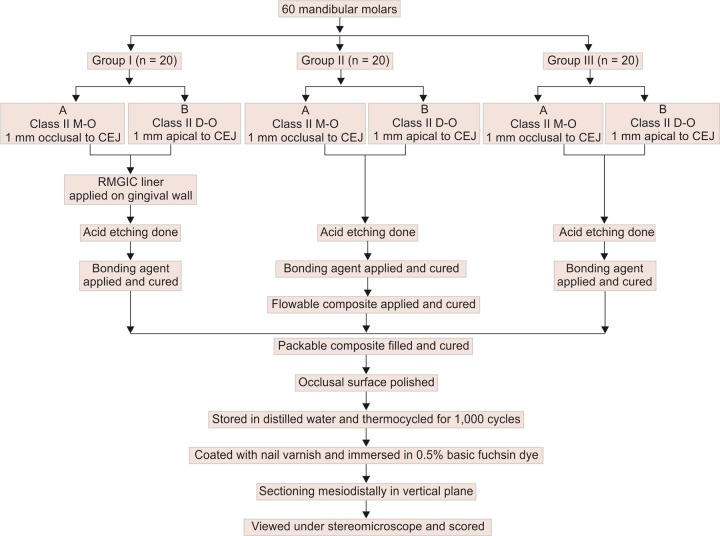
Methodology (procedural sequence)

Results in this study, showed that when flowable liner was placed apical to CEJ (group IIB) showed least marginal leakage when compared to other groups on distal side. These results are in agreement with the results of study of Leevailoz C et al who concluded that gingival margins (dentin) had higher microleakage than occlusal margins (enamel) and flowable composite helped in reducing microleakage at the gingival margins (apical to CEJ) when placed below packable and microhybrid resin composite, in class II restorations. With regard to clinical concern, results of *in vitro* studies are often presumed to be more negative than *in vivo* studies, suggesting that leakage found *in vitro* should be regarded as a theoretical maximum amount of leakage that may or may not occur *in vivo*.^16^

## CONCLUSION

Finally, result of the present study indicates that there is less microleakage in enamel margin than dentin margin and there is no need for placing liner below class II, because placing liner does not have significant effect on microleakage when margins were placed in enamel. The use of flowable composite and RMGIC, as a liner can significantly diminish microleakage along the dentinal gingival margin and this technique could therefore be considered as a viable modality in class II composite restoration when margins are placed apical to CEJ, in the dentin.^17^

There are no accepted scientific methods to correlate *in vitro* leakage results to clinical findings. Hence, further clinical trials are essential to know the *in vivo* variables which could affect the outcome of this study.
